# The political economy of health financing reforms in Zimbabwe: a scoping review

**DOI:** 10.1186/s12939-022-01646-z

**Published:** 2022-03-27

**Authors:** Alison T. Mhazo, Charles C. Maponga

**Affiliations:** 1 Ministry of Health, Community Health Sciences Unit (CHSU), Private Bag 65, Area 3, Lilongwe, Malawi; 2 Department of Pharmacy and Pharmaceutical Sciences, Faculty of Medicine and Health Sciences, University of Zimbabwe, P. O. Box A178, Avondale, Harare, Zimbabwe

**Keywords:** Political economy, Health financing reforms, Universal health coverage, Health equity, Ideas, Institutions, Interests

## Abstract

**Background:**

Implementation of health financing reforms for Universal Health Coverage (UHC) is inherently political. Despite the political determinants of UHC, health financing reform in Zimbabwe is often portrayed as a technical exercise with a familiar path of a thorough diagnosis of technical gaps followed by detailed prescriptions of reform priorities. In this study, we sought to understand the interaction between political and economic aspects of health financing reforms since the country got its independence in 1980.

**Methods:**

We conducted a scoping review of health financing reforms in Zimbabwe and reviewed 84 relevant sources of information. We then conducted a thematic analysis using an adapted Fox and Reich’s framework of ideas and ideologies, interests and institutions.

**Results:**

We found that ideas, institutions and interests significantly influence health financing reforms in Zimbabwe with implications on health system performance. Reform priorities of the 1980s were influenced by socialist ideologies with an interest to address pervasive health inequities inherited from the colonial racial system. The progress in equity realized in the 1980s was severely disrupted from the 1990s partly due to neo-liberal ideologies which steered interests towards market-oriented reforms. The period from the 2000s is characterized by increasing donor influence on health financing and a cumulative socio-economic collapse that resulted in a sharp and protracted decline in health spending and widening of health inequities.

**Conclusion:**

Health financing reform process in Zimbabwe is heavily influenced by political economy characteristics which favor certain financing arrangements over others with profound implications on health system performance. We concluded that the political economy factors that slow down UHC reforms are not rooted in the ambiguities of ideas on what needs to be done. Instead, the missing link is how to move from intention to action by aligning espoused ideas with interests and institutions which is an inherently political and redistributive process. International and domestic actors involved in UHC in Zimbabwe  need to explicitly consider the politics of health financing reforms to improve the implementation feasibility of desired reforms.

## Background

Globally, health financing has emerged as an attractive reform area for Universal Health Coverage (UHC). UHC stipulates that people should access the health services they need without risking financial ruin or impoverishment [1], a concept that Zimbabwe subscribes to. Features of the health financing system that hamper progress towards UHC in Zimbabwe have been previously noted with accompanying recommendations for requisite reforms [2, 3]. In the case of reform processes in Zimbabwe, a historical and longitudinal perspective is particularly important considering that the country has gone through multiple transitions that were intertwined in a not always mutually supportive way in what has been called ‘a transition overload’ [4]. Rooted in UHC is the aspect of health equity which is defined as the absence of systematic disparities in health (or its social determinants) between more and less advantaged social groups [5]. Health equity is a multidimensional concept that has been internationally considered as an essential element for health system development, including in Zimbabwe. The enduring importance of health equity in post-independent Zimbabwe is demonstrated by its dominance in all major health policies and strategies since 1980. Despite the aspiration, inequitable access to health care remains one of the most pressing challenges and out of pocket expenditure continues to be an important source of financial hardship [3, 6].

From an ideological standpoint, health equity is not a technical aspiration but rather an ethical concept based on the principles of distributive justice and human rights [5]. Thus, tackling inequities in health has been a longstanding priority for societies, considered as a moral intervention to create an even society. In a widely cited 1992 paper on the concepts and principles of equity in health, Whitehead defined health inequities as differences in health that are unnecessary, avoidable, unfair and unjust [7]. The organization and management of the health financing system exerts a considerable influence on health equity. Consequently, health financing reforms have been framed within the aspiration of addressing health inequities. Health financing reform is an inherently political process that alters the distribution of entitlements, responsibilities and resources across the health sector and beyond [8]. By altering incentives and creating losers and winners, health financing reform fits the 1936 seminal definition of politics by Harold D. Lasswell who defined it as a competition about 'who gets what, when, and how' [9]. Thus, health financing reform is not a technical exercise, it is rather an arena of political competition through a complex interaction of ideas, institutions and interests with contested arguments and diverse stakeholders capable of facilitation or resistance—even veto policy options [10, 11] . Despite the importance of assessing health financing reforms through a political economy lens, health financing reform in Zimbabwe is often presented from a technical perspective along a familiar pattern of detailed diagnosis of the health financing ‘gaps’; followed by a catalogue of ‘prescriptions’ on how to progress towards UHC. We argue that by over-emphasizing this techno-centric view of health financing reform whilst sidelining the broader political economy in which the reforms are intended to occur, the viability of proposed interventions becomes severely challenged. Political economy is the study of both politics and economics and focuses on power and resources, how they are distributed and contested in different country and sector contexts, and the resulting implications for development outcomes [12]. In this study we conducted a scoping review of literature and applied a political economy lens to health financing reforms in Zimbabwe, an approach that has recently received international attention for its usefulness in developing strategies to change the political feasibility of pro-UHC reforms [8].

The overall goal of the study was to analyze how the interplay of ideas, institutions and interests prioritized certain health financing reforms over others. The specific objective was to develop an analytical view that could be used as a guide to understand the political economy of health financing reforms in Zimbabwe, thereby anticipate the conditions under which UHC reforms are likely to thrive or fail. In so doing, proponents of health financing reforms in Zimbabwe could prospectively manage reform processes and enhance their technical and policy feasibility. To guide our analysis, we used the Ashley M. Fox and Michael R. Reich framework (2015) for analyzing the politics of UHC reforms in Developing Countries [13]. The framework analyzes the politics of health reform for universal health coverage according to four stages in the policy cycle (agenda setting, design, adoption, and implementation) and four variables that affect reform (interests, institutions, ideas, and ideology). Below we present the four variables that affect reform.

### Ideas and ideology

Ideas include specific policy solutions, information, and prevailing concepts and paradigms that influence thinking on a subject [13]. On the other hand, ideology’ is the higher-level political framing against which ideas are explored and establishes the political rationale and justification for the adoption and implementation of UHC. Ideology represents a particular worldview, usually presented along a continuum of beliefs and philosophical inclinations. Incorporating ideology into the analysis of health financing reforms is critical because ideology particularly affects the progressivity of financing structures and the degree to which the private versus public sector is preferred as the vehicle for service delivery [13]. By emphasizing ‘access to care based on need and not ability to pay’, UHC is rooted in the egalitarian concept (ideology) which is focused on the equality of every individual. The characterization of inequities as ‘*unnecessary, avoidable, unfair and unjust ‘*also carries an ethical attribute that is driven by ideological inclination towards social justice. In sum, ideas are powerful, because they embody the narratives, the metaphors that shape how UHC is perceived, and how it is discussed and popularly represented. The power of the ideas rests on the notion that symbols or a persuasive story can be more important than material or objective fact [10, 14].

### Institutions

Institutions encompass both a country’s formal political institutions that affect how policy is made and its informal institutions. This includes the legacies of past policies or even cultural norms embedded in how policy decisions are made [13]. Countries with institutional designs that incorporate a greater number of “veto points”— where the consent of certain individuals or bodies is required to pass legislation— will experience more difficulties in passing health reform options [10, 15, 16]. A key feature of institutions is path dependency, which in most general form, simply claims that the past has a powerful effect on the present which creates a historical policy legacy [17]. These historical legacies “lock” countries into certain trajectories and exert inertia on current politics even after a long time lag and act as potent focusing devices that constrain future policy changes from the status quo. This 'lock in' effects occurs  even if the initial choice was suboptimal [18].

### Interests

Interests include all stakeholders who will benefit or be hurt by a given policy (winners and losers). Interest group theories explain policy change (or the lack of policy change) as emanating from the relative power balance and intensity of position of different groups of stakeholders who will be affected by reform efforts [13]. Analysis of interests therefore emphasizes key elements or explanatory variables that examine the role of actors and power in policy reform [19]. Common actors involved in health financing reforms include Ministries of Finance, provider associations, insurance companies, donor agencies, progressive social movements, and advocacy groups.

## Methods

We conducted a scoping review using the Arksey and O’Malley framework [20]. At a broader level, a scoping review aims to map rapidly the key concepts underpinning a research area and the main sources and types of evidence available. We followed the next steps for conducting the scoping review: identifying the research question, identifying relevant studies, study selection, charting the data, collating, summarizing, and reporting the results.

### Research question

Our research question was: What is the influence of ideas, institution, and interests on health financing reforms in Zimbabwe?

### Identifying relevant studies

We first conducted a search of electronic data databases: PubMed, Medline, Academic Complete and WHO Medicus. Since various aspects of health financing and health performance in general are often reported together, we used a variety of search terms including those related to efficiency to initially cover breadth. Using our pre-existing knowledge on the health system in Zimbabwe, we used the combination of search terms below.

*Health financing OR health insurance OR primary health care or universal health coverage or Donor funding.*

*AND*

*user fees, AIDS levy, Results Based financing, structural adjustment program, equity, utilization, out of pocket, efficiency, expenditure, health insurance, medical aid society, reimbursement, universal health coverage, primary health care, collection, pooling, purchasing, subsidization, catastrophic health expenditure.*

*AND*

*Zimbabwe.*

Use of generic terms was deliberate as we sought to find as many articles as possible to cover breadth. An initial search in PubMed, Medline, Academic Complete and WHO Medicus generated a total of 2482 articles. This high output could have been driven by the generic terms that were used for the initial search. EndNote Online and EBSCOhost databases were used to organize the literature.

### Study selection

**Fig. 1:**
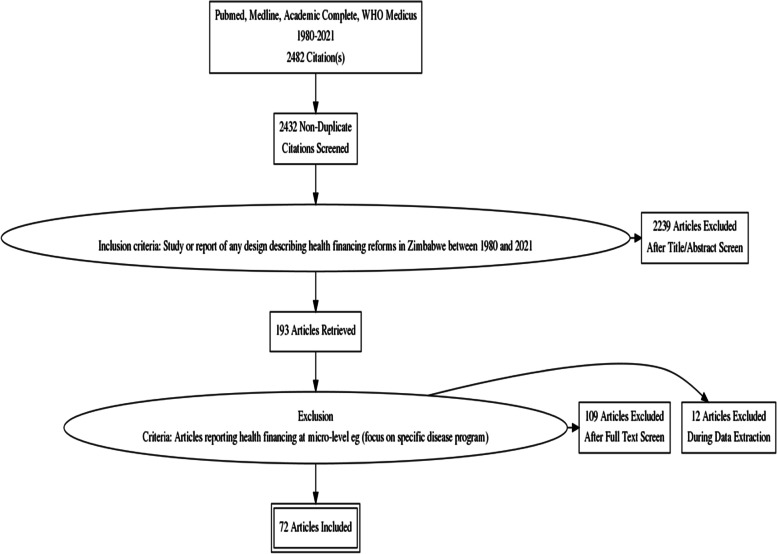
PRISMA Flow diagram

After removing the duplicates, our inclusion criteria was any study or report of any design that describes health financing reforms in Zimbabwe between 1980 and 2020. This broad inclusion criteria in terms of study design and period was deliberate for three reasons: 1) our initial presumption was that there could be not so many articles that specifically address health financing reforms in Zimbabwe. Therefor, a trict eligibility criteria could have resulted  in ‘an empty review’ 2) health financing reforms are system interventions where studies may not fit neatly into the archetypical hierarchy of evidence where clinical trials are ‘gold standard’. Therefore, there were a wide variety of study designs that could still deepen an understanding of the political economy of reforms without strictly conforming to typical study designs viewed as robust from a medical science perspective. Notably, since we were interested in ideas (ideology), we found debate-focused articles to be particularly useful as sources of arguments for or against major health financing reforms 3) the studies covered a longer period (since Zimbabwe got its independence in 1980 to 2020) because we wanted a longitudinal and historical perspective since. We therefore conceived that analyzing health financing reforms cross-sectionally would overlook how the structure of a given system, in terms of its financing and infrastructure, determined the scope for the successive evolution of health systems [21]. After applying the inclusion criteria, 2239 articles were removed after title and abstract screening resulting in 193 articles remaining. We did not specifically look for the word health financing’ or any of its derivatives in the title or abstract based on our pre-existing knowledge that health financing aspects  could be reported in articles that focus on general health system performance. We perceived a review of 193 articles to be time-consuming and applied a subsequent selection criteria where we excluded articles that focused on health financing reforms at micro-level since our interest was on system (macro) level reforms. We defined micro-level as articles that focused on a particular disease or condition which was dominated by HIV/AIDS, cervical cancer and maternal and child health. An exception was made when reform efforts aimed at a disease or condition elicited system changes. We therefore included reform efforts for HIV/AIDS (the creation of the National AIDS Trust Fund) and maternal and child health (purchasing reforms in the form of Results Based Financing). A total of 109 articles were removed for reporting micro-level aspects; remaining with 84 articles eligible for full text screening. Out of these, 12 articles published between 1981 and 1982 could not be reviewed because we failed to retrieve the full articles. A final list of 72 articles was eligible for full review. Additional 12 articles were obtained from the references of eligible articles and purposive google search of key reports and policy documents based on our pre-existing knowledge of Zimbabwe’s health policy environment. Figure 1 below shows the PRISMA Flow diagram for the study selection process.

### Data charting

After identifying eligible articles, we conducted data charting. Data charting is a technique for synthesizing and interpreting qualitative data by sifting, charting, and sorting material according to key issues and themes. First, a data charting tool was developed in Microsoft Excel. Next, we categorized the contents of each eligible article according to the elements of the Fox and Reich framework-institutions, interests, and ideas. Data charting involved an iterative process of interpretive data analyses and refining. The first step involved verbatim extraction of text excerpts from the selected articles which were categorized according to the components of the data charting tool. The second step involved summarizing the contents and some re-categorization where the misclassification was observed from the initial categorization.

### Findings

The findings of the analysis have been organized using the Fox and Reich framework and summarized in the table below.

### Ideas and ideology

The ideas and ideology influencing health reforms in Zimbabwe have gone through evolutionary cycles in light of the  shifting political and economic contexts. The first decade post-independence  (1980-1990) was driven by the idea of nation-building, equality, and solidarity as a post-colonial strategy [22–24]. Central to this thrust was the idea of disrupting the pervasive social injustice that prevailed during the colonial period where a racially divided health care system accorded the minority White population sophisticated health care amenities whilst the majority Black population remained marginalized and plagued with diseases of poverty [25, 26]. Whilst the colonial government had never published health indicators by race, the newly independent government released statistics that showed that the expenditure per capita was 36 times higher amongst White population compared to the rural Black population [27]. Infant mortality rates, arguably the single most important public health statistic, also showed marked differences across racial groups. At independence, Whites were estimated to have an infant mortality rate of 12 per 1000 live births; urban Blacks, 60 per 1000 live births; and rural Blacks, 120 per 1000 live births [26]. The disparities were so wide but of no consequence to the colonial administration to the extent that when the pattern of morbidity among Europeans in the then capital of Salisbury (now Harare) were being examined, reports of outpatient care in England were used as the comparison [26]. In response, the government implemented policies aimed at tackling racial inequalities inherited from the colonial period within the Primary Health Care (PHC) concept [28, 29]. In a White Paper entitled *Planning for Equity in Health, A Sectoral Review and Policy Statement* the government of Zimbabwe detailed how it intended to transform the existing health care system into one consonant with the government’s commitment to socialist development [25].

Towards the end of the 1980s, the country started experiencing fiscal deficits compounded by intermittent droughts, global economic recession, and a slump in commodity prices [30–32]. Driven by a global wave of economic recession and fears of unsustainability in the patterns of social spending in Low-Middle-Income Countries (LMICs), international institutions promoted the idea of economic growth fueled by increasing exports, trade liberalization and diminished social subsidization [33]. This neo-liberal ideology -which celebrated the power of markets and framed public sector as ‘inefficient’- infused ideas towards efficiency and private sector models in service delivery [34, 35]. Interestingly, Zimbabwe was profiled as an exemplar global model in the 1987 World Bank report that advocated for sweeping reforms in the financing of the health sector in LMICs entitled *‘Financing health services in Developing Countries: An agenda for reform’*. The re-orientation towards market ideals also brought changing ideas about the management of the public sector, in particular stemming from new public management theory that was making tide across USA, Canada and Europe [34–36]. At the core of this ideological inclination was an ambivalent view on the role of the state in service provision which was framed as ‘inefficient’. The most controversial and arguably the most profound idea brought by the ascendancy of neo-liberal ideology was the introduction of user fees in health care. In 1992 and 1993, the World Bank released two separate reports that provided reform strategies for strengthening the user fee system in Zimbabwe [37, 38]. The idea underpinning user fees was that individuals should contribute to health care since public spending was not sustainable and inefficient. In essence, the shift from an expansionary, publicly funded health system to a contractionary model based on user fees was a reversal of the policy on access to health care based on need to access based on the ability to pay. Ideologically, by levying the sick to access services, the policy was a burden on the heavy users of care and a direct contradiction to the egalitarian tenets of solidarity and cross-subsidization. Thus, user fees –driven by the neo-liberal ideology- severely regressed the equity gains realized in the 1980s [22, 24, 39–41].

Although user fees are often labelled as a part of a ‘prescription’ of structural adjustment programs imposed by international institutions, and often blamed for the ‘marketization of health services’, it is worth situating them within the broader political economy realities of Zimbabwe. Through a technical interpretation of the performance of Zimbabwe’s economy put forward by a team of technocrats assembled by the then Minister of Finance Bernard Chidzero, the group convinced the ruling party that the  Economic Structural Adjustment Program (ESAP)  was necessary. The raft of neo-liberal policies that accompanied  that drastic policy shift  from state-led policies to pro-market ideas has been dubbed as the genesis of the ‘decline of socialism’ in Zimbabwe [42]. Other scholars argue that the adoption of ESAP and its attendant policies such as user fees was a sign of a decline in the degree of priority attached to the absolute welfare of the poor [43].

The twenty-year period from 2000s to 2020 is characterized by increasing influence of the donor community in response to two major reinforcing crises: an epidemiological crisis induced by HIV/AIDS in the 2000s and a precipitous economic decline that started from 2000 and peaked into a crisis around 2008. The framing of HIV/AIDS, TB and Malaria control as a developmental strategy under the Millennium Development Goals (MDGs) and the 2001 United Nations General Assembly Special Session (UNGASS) Declaration of Commitment on HIV/AIDS had a profound effect on the funding architecture for Zimbabwe’s health sector. Despite hostile donor relations and dwindling external support, Zimbabwe received vertical funding from the Global Fund to Fight AIDS, Tuberculosis and Malaria, the Expanded Support Programme (a basket-funding mechanism supported by several countries), and the United States Presidents Emergency Plan for AIDS Relief (PEFPAR) [44]. The socio-economic threat of HIV/AIDS and the uncertainty of donor support in the midst of political tension led to the creation of the National AIDS Trust Fund (NATF), an earmarked, payroll-based funding dedicated for a multi-sectoral response to HIV/AIDS. The NATF has acclaimed exemplary status as an innovative domestic financing model for HIV/AIDS in sub-Saharan Africa [45, 46]. In the aftermath of the cumulative socio-economic breakdown that severely disrupted the quantity and quality of health services in the late 2000s, the ideology and ideas on health financing were re-oriented from an epidemic focus of the early to mid-2000s to an emergency recovery mode. As a result, the environment was receptive to health financing ideas that were both ‘unconventional’ and typical of fragile and conflict affected states [47–49]. An archetypal example is the introduction of purchasing reforms in the form of Results Based Financing (RBF) for maternal and child health in 2011.

The idea of RBF was not completely novel in Zimbabwe as it ‘retrofitted’ with the Results Based Management (RBM) that was conceived by the Ministry of Health in 2004 but could not be implemented due to resource constraints [49]. Ideologically, RBF is in tandem with the ideas of ‘new public management’ which emphasize ‘management by results’ [50]. The characterization of Zimbabwe as a fragile state also reinforced the idea of enhancing donor aid effectiveness in line with the principles of the 2005 Paris Declaration on Aid effectiveness that emphasized aspects such as transparency, harmonization and country ownership [48]. Alongside these donor funded initiatives, in 2017 the government introduced a 5% Mobile Airtime Levy (also known as health levy) ring fenced for procurement of medicines.

### Institutions

During the colonial period, institutions were designed to entrench racial inequalities between Whites and Blacks. The Medical Services Act of 1979 allowed certain beds in the best government hospitals to be ‘open’ to designated medical practitioners to admit their private patients, while the African wards were ‘closed’ to private doctors [25]. Additionally, the levying of user charges discriminated the population according to ability to pay which further entrenched the segregation. The quality of care between open and closed wards was markedly different and permeated every facet of treatment and care including food, drugs, and amount of nursing (including the racial composition of nurses). The allocation of health funding was also biased towards large, curative biased urban centers that mainly served the White population. Buoyed by a revolutionary mandate, the ruling party intended to de-racialize the institutions inherited from the colonial period and pursued broad-based welfarist policies particularly on education and health. This was facilitated by centralized state planning and a top-down approach to resource allocation inherited from the colonial administration. A key institutional reform effort was aimed at the integration of the fragmented health care system and facilitation of community participation in the health system. Thus, at the base of the health service was the Village Health Worker (VHW), through whom democratization of the health system was to be attained [24].

Institutional reforms aimed at expanding the sources of revenue and forms of health financing mechanisms also entered the policy domain in the mid-1980s. These included reforms emphasizing pre-payment financing methods as espoused under the contemporary UHC discourse. In a 1984 White paper entitled *‘Equity in health planning’*, the government pronounced the intention to create the National Health Service that would be financed out of central government revenue. It also proposed funds to be raised through a scheme involving compulsory national health insurance as part of the National Social Security System that was due for adoption [25]. In 1992, the World Bank underscored the creation of the National Health service and went further to propose the establishment of a National Health Development Fund (NHDF) incremental to the MOH budget [37]. Analogous to what is now termed ‘sin tax’ that is being promoted as one of the diversified means of raising funds for UHC [51, 52], a proposal was made to fund the NHDF through taxes on alcohol or tobacco. The NHDF was intended to be earmarked for essential primary care and preventive health services, especially in poor, underserved rural and urban communities. The first public social security institution- National Security Services Authority (NSSA) -was established in 1993 without the component of the compulsory health insurance [53]. In the 1990s, the introduction of user fees and infusion of market ideas in the health sector steered health system governance towards decentralized decision making. Under the Bamako Initiative introduced by UNICEF and the World Health Organization in 1987, a key principle was to decentralize retention of user fees to the local level in health centers managed by a committee of community representatives. Zimbabwe introduced the Health Services Fund (HSF) in 1996 to expand and improve service delivery through decentralized funding to district hospitals and rural health centers. The HSF involved collection of user fees at the point of service use and local retention to cover non-salary recurrent expenditure that included the cost of medicines. In anticipation of the harmful impacts of ESAP on the poor, the IMF advised the introduction of Social Dimensions Fund (SDF) to cushion those affected with structural reforms, a package which included an exemption for user fees amongst eligible groups [54]. To promote efficiency, commercially oriented mechanisms of health sector regulation were introduced including contracting out in line with the principles of Public-Private Partnerships [35, 55].

The 2000s began with a radical policy of land redistribution followed by an immediate fall out with Western countries over alleged missteps in governance. In response, Britain, the European Union (EU), and some Commonwealth countries imposed various forms of ‘targeted’ sanctions on Zimbabwe. The EU in particular adopted a common position to terminate official development assistance directly to the Zimbabwean state and redirected aid, including for health to non-state organizations [56]. In 2001, the USA enacted the Zimbabwe Democracy and Economic Recovery Act (ZIDERA) with a key provision for restricting the USA from voting in support of new assistance to Zimbabwe from international financial institutions except for programs that meet basic human needs or promote democracy. This environment of deteriorating relations with erstwhile Western donors was characterized by a massive erosion of trust towards Zimbabwe’s central government and its institutions. In a sign of trust breakdown, donors started bypassing the government and channeled their funds through various non-state actors including United Nations (UN) organizations and Non-Governmental Organizations (NGOs). After the 2008 economic collapse, the formation of a Government of National Unity in 2009 coincided with immediate influx of Western funds, including from EU and its member states which provided more than US$2 billion for assistance to education, water, sanitation, health, agriculture and food aid [30].

In 2010, the Health Sector Investment case was launched with the aim of moving from an emergency planning mode to a stability phase with a number of pledges from international donors. A number of institutional reforms came out of that renewed interest in the health sector. In a volatile social, economic, and political context characterized by deteriorating health indicators of humanitarian proportion, the Health Transition Fund (HTF) was designed as a multi-donor pooled fund aiming to support the government in inverting trends in maternal and child health with an allocated investment of US$235 million. The UK Department for International Development (DFID), the EU, the Governments of Ireland, Sweden, Norway and Canada were the major contributors to the fund with UNICEF as the fund manager [48]. Architects of pooled donor funds claim that the model might not dominate aid flow at country level but can significantly alter institutions since the mechanisms and processes involved (particularly joint planning and country ownership) can trigger new ways of donor collaboration in the wider environment. Under similar context, the World Bank offered a $US 15 million grant, which was conditional on using the RBF mechanism for maternal and child health. As Zimbabwe was in arrears with its inter-national debt payments, it was not eligible for regular International Development Association loans, and so the Health Results Innovation Trust Fund, which supports RBF approaches, was the only funding vehicle the World Bank could offer [49]. Similarly, due to legacy international arrears and the attendant adverse lending conditions, the World Bank could not directly allocate the fund to government and third parties were contracted to manage the funds. Thus, RBF brought new institutional arrangements in the form of ‘verification ‘of outputs at government facilities through third party agents, direct payment of incentive bonuses to facility staff based on performance and changes on financial management rules. Opening of bank accounts by facilities and greater autonomy to use funds was a shift from the legacy policy under the Health Services Fund where facilities held virtual accounts at district level since 1996 [49]. The various institutions introduced following the collapse of the health system are still in place, albeit with successor plans and some structural changes but the central argument or ‘idea’ underpinning their establishment has largely  been retained.

### Interests

In 1980, the government of newly independent Zimbabwe had very clear interests informed by a century of colonial domination and White privilege. At the core of the inequities was a parallel developmental strategy premised on the apartheid-type logic of separating racial groups into non-competitive social, economic, and political systems [57]. Under the policy of *‘Growth with Equity’*, the national democratic phase enunciated the following interests: to narrow the economic gap between racial groups by redistributing some of society’s assets and incomes, revitalizing existing African assets, and providing new, non-material assets to Africans in the form of improved education and health facilities [57]. In line with this thrust, the government pursued a well-articulated and elaborate agenda to dismantle inequities particularly for health and education [39]. Embedded in the approach was the urgency to correct the structural determinants of uneven development emanating from a colonial history and a capitalist economy that differentiated the country into urban and rural areas. Whilst the first decade post-independence is dominated by a public policy discourse of pursuing broad-based socio-economic reforms to remedy the colonial legacy of racial injustices, critics have highlighted some salient governance dynamics that presented a paradox from a broader political economy perspective. The first criticism is that the post-independent period coincided with exclusionary developmental politics characterized by regional marginalization for socio-economic development including for health [58]. The second criticism is that an anti-developmental patrimonialism ensued as elite coalitions opportunistically extracted economic rents through a social order that relied on patronage relationships [59].

On top of the broader governance dynamics that influenced socio-economic development, the government also faced important policy trade-offs to balance public and private sector interests. At independence, Zimbabwe inherited a sophisticated private sector that was developed to serve the affluent minority White population comprising a medical sector and health insurance (referred to as medical aid societies). In practice, the medical sector basically comprised of physicians practicing privately and receiving fee for- service from medical aid societies or directly from patients [25]. In light of the contradictions between the reality of private sector dominance and its vested interests viz-viz the government’s vision of a socialist health care system, it was often suggested that the litmus test for the government's commitment towards a socialist health model was its attitude and policies towards the private medical sector. The government intervened to slow down the growing dominance of the private sector; including a ministerial decree to stop the construction of three private hospitals [25]. On the other hand, the private sector employed a strategy by offering a package of health care services previously denied to the urban blacks and the affluent blacks (those in the ‘economically viable sector’) in order to diffuse the political support and momentum for a national health service [25]. Whilst this strategic counter-maneuvering was playing out, underpinning the government’s cautious approach to regulate the conduct of the private sector (dominated by Whites) was the policy of racial reconciliation that was adopted at independence [24].

From the 1990s, actors with interests in globalization and neoliberal approaches to development in particular the World Bank played a dominant role in reconfiguring economic, social, political, and institutional relationships including for health [60]. To illustrate the growing importance of health to the World Bank, its 1993 world development report was devoted to health issues in developing countries entitled *‘World Development Report 1993: investing in health’* [61]. In addition to its global influence as a source of ideological shifts in LMICs, the World Bank also allocated huge amounts of money for preferred policy options, often attached to aid and loan conditionalities amongst aid-recipient countries [62]. For example, between 1992 and 1996, the World Bank and other multilateral and bilateral donors allocated $120 million to Zimbabwe for implementation of the Second Family Health Project, designed to directly benefit low-income households likely to be affected by spending cuts influenced by structural adjustment programs, especially women and children [61]. The World Bank also promoted expansion of the private sector. In its 1992 health policy reform paper for Zimbabwe, it proposed the expansion of the private sector (private medical sector and private health insurance). Although many scholars and critics label the introduction of market-oriented reforms in LMICs as *‘prescriptions’* to portray imposition of the ideas on acquiescing individuals and institutions, there was visible form of contestation in Zimbabwe. For example, the then Minister of Health Dr. Timothy Stamps publicly criticized the reforms and expressed critical skepticism on the merits of the 1993 World Bank development report [63].

The period from 2000s to 2020 was characterized by the entry of new actors in response to the HIV/AIDS, TB, and Malaria epidemics. Later on this included actors who had an interest in avoiding a humanitarian catastrophe after the 2008 economic collapse. In response to the epidemics, some of the major actors that came into the picture included the Global Fund to Fight AIDS, TB, and Malaria (Global Fund), PEPFAR, United States Agency for International Aid (USAID) and United Kingdom Department for International Aid (DFID). Globally, HIV/AIDS ‘democratized’ health systems and allowed the participation of new actors as affected communities self-organized to address their own interests particularly stigma and discrimination. Underlying the approach was the perceived passive response to HIV/AIDS on the part of governments [64]. The idea gained traction after the United Nations General Assembly Special Session on HIV/AIDS (UNGASS) which was held in 2000 with a declaration for public participation and civil society as important elements of a global alliance to eliminate HIV infections. This culminated in ideas such as ‘Nothing about us without Us’ to reflect demand for inclusion in the design of HIV/AIDS  programs [65]. Community and activist organizations were among the first to alert people to the realities of the epidemic and to pressure government to place treatment on national health agendas in Zimbabwe [66]. Whilst the involvement of communities had been at the center of health programs in line with PHC, organizations such as the Global Fund made it a requirement for countries to demonstrate inclusion of those disproportionately affected by HIV/AIDS or key populations ‘to sit on the table’ when funding and programing decisions were being made. Of note was the formal engagement of previously marginalized groups such as sex workers. The Global Fund also created platforms for affected populations to foster government accountability in the provision of services through structures such as the Country Coordinating Mechanism (CCM). Table 1 below summarizes the major health financing  reforms in Zimbabwe  according to the political economy factors from 1980-2020.

**Table 1:** Major health financing reforms and associated political economy factors

Period	Major Reform (s)	Ideas	Institutions	Interests
1980–1990	Primary health care, comprehensive and macro-level state driven health financing	-Evidence of racial imbalances in healthcare -Wide disparity of access between the White minority and Black majority -Primary health care -Massive rural-urban divide in access to health care	-A centralized state -Socialist/nationalist ideas -Favorable attention in the international community -Inheritance of a well-organized and sophisticated private care to serve the Whites -Inheritance of pro-urban and large facility bias in allocating funds -Economic recession, droughts and inflation in the late 1980s raised threats of unsustainability in public funding for health	-Equity -Fulfilling liberation war promises on the part of the state -Rising middle class interested in accessing privileges formerly reserved for Whites under minority rule -Preservation of private sector arrangements
1991–2000	-User fees -Macro level intervention	-Unsustainable rise in health expenditure -Inefficient public sector -Market principles for the public sector	-Increasing influence of the multilateral financiers on state administration -Globalization	-Efficiency -Reduce public subsidies and expenditure on health -Raise revenue -Reduce unnecessary health consumption
2001–2010	-Vertical funding of TB, HIV/AIDS and Malaria by international donors -Earmarked funding for HIV/AIDS	-TB, HIV/AIDS and Malaria a threat to global health security -The three epidemics a major obstacle for national and global development in the context of Millennium Development Goals -The economic threats of HIV/AIDS	-International isolation of Zimbabwe due to alleged human rights abuses -Economic contraction from 2000 and collapse around 2008 -Dramatic decline in health expenditure -Severe deterioration in health delivery -Influx of earmarked donor funds -Donor funds channeled through non-governmental channels -Creation of the National AIDS Trust Fund (NATF) through the National AIDS Council (NAC)	-Influx of earmarked donor funding for TB, HIV/AIDS and Malaria -Global Fund and PEPFAR -Donor funding provided as humanitarian aid not developmental aid
2011–2020	-Targeted pooled donor financing post crisis and selected purchasing reforms -Mobile airtime levy earmarked for health financing	-Zimbabwe perceived as a fragile country post a decade of socio-economic meltdown -Evidence of worsening health indicators particularly affecting women and children	-Warming up in donor relations after the Government of National Unity -World Bank grant on conditionality of implementing Results Based Financing (RBF) -Institutions found to retain some resilience despite years of underfunding and not consistent with a fragile environment -Quick impact of RBF attributed to historical legacy of strong institutions put in place in the 1980s -General macro-economic stabilization	-Immediate recovery effort for the health sector -Improve the health of women and children -Results Based Financing

## Discussion

The sections below discuss the interaction of the political economy factors and the comparison of Zimbabwe and other settings**.**

### Interaction of ideas, institutions and interests and the policy cycle stages

From a political cycle of policy reform perspective, broadly speaking the early 1980s coincided with the ascendancy of universal health care onto the political agenda. A policy agenda is defined as a the list of subjects or problems to which government officials, and people outside of government closely associated with those officials, were paying some serious attention at any given time [67]. Drawing from Kingdon’s Multiples streams model [67], Fox and Reich put forward that UHC appears on the agenda when different streams come together at the right moment— a convergence of an ongoing problem with a political window (occasioned by political and economic transitions) and an available policy solution. Universal health care rose to the agenda in the 1980s with the main interest to address the problem of health inequities driven by a colonial legacy of racial discrimination. The political transition to majority rule provided a window of opportunity to advance an ideology of nation-building and solidarity. That in turn steered institutions towards a PHC approach under a socialist model. There was also a global window of opportunity since Zimbabwe got its independence one year after the Alma-Ata Conference on PHC which emphasized ‘Health for All by 2000’. The PHC concept  had its roots in the ideology of de-colonization, global solidarity and equity [68, 69]. The favorable ascendancy of PHC to the agenda was also driven by war-time experiences where freedom fighters had attributed the prevalent of preventable deaths to the ‘ignorance of primary health care’. This had led to the establishment of a department of primary health care to address the problem at the height of the liberation struggle [70].

The design of PHC in Zimbabwe was influenced by domestic ideas on how to address the problem of inequities in health whilst  providing an optimal mix of services. Three ideational inclinations influenced the design of the PHC model and the policies that were socially acceptable from a political economy perspective: 1) tackling the disparities between the rural and urban population 2) tackling inequities between the private and public sector and 3) trade-off between curative and preventive services. A comprehensive approach that incorporated these ideas led to impressive improvements in essential health indicators [24].

### Comparison of political economy variables between Zimbabwe and other settings

This study examined in detail the historical influence of ideas, institutions, and interests in shaping health financing reforms in Zimbabwe. An important finding coming out from our analysis is that political economy factors that influence reform processes were highly context specific. This is in corroboration with other studies [8]. Despite the context-specific nature of the reform process, we observed that political econmy analyis (PEA)  for health reform and the resultant frameworks were mainly viewed within the lens of developed countries. We argue that whilst the use of these  frameworks is highly valuable, their blunt application might potentially obscure the salient features that uniquely shape reform processes in a developing country such as Zimbabwe. To illuminate some of these differences, we used the Fox and Reich framework to draw some comparisons that carried conceptual and practical implication in approaching PEA in Zimbabwe.

Regarding ideas/ideology, the PEA of health financing reforms in developed countries and some middle-income countries reveal the visible influence of contesting ideologies, typically referred to as right-left wing politics [8, 71, 72]. International experience shows that left-wing parties tend to enact redistributive policies such as UHC [73–75]. However, these outright (and at times polarized) ideologies were not clear cut in the Zimbabwean context. Although  we identified a clear socialist ideology in the 1980s, which could be viewed as extreme leftist, there was no visible contestation that originated from a counter-veiling ideology. The fundamental difference stems from the fact that  PEA in developed countries and some middle income countries rests on an assumption of a pluralistic society where power is dispersed amongst stakeholders [76]. As illustrated by the USA affordable care act, the PEA for health financing reforms often reveals open contestation amongst interest groups such as trade unions, medical providers, medical insurance and pharmaceutical industry as each group calculates the likely impact of such reforms [16, 77]. The political history of some legacy social health insurance systems that are being promoted as a viable pathway to achieve UHC also shows this open contestation. For example, the first system of organized health care emerged in Germany in the nineteenth century as part of a series of social reforms introduced by Bismarck to quell political incursions by the social democrats and labor unions [72]. However, in our study, we found that Zimbabwe tended to have a very centralized system of governance and dominantly state led-policies. As a result of these governance dynamics, health reforms were not routinely subjected to open scrutiny and competition amongst potential interested parties and well organized policy networks particularly at the agenda setting stage. However, as highlighted before, some contestation was visible for HIV/AIDS policy making and financing within the discourse of human rights and the influence of external agencies. The apparent homogeneity in ideology and non-visible competition of interests carried important implications regarding the role of institutions in mediating reform processes in Zimbabwe. Notably, whilst in some developed countries health financing reform is through a legislative process which provides multiple ‘veto points’ [15, 16], this was not clear cut in Zimbabwe. Therefore, whilst policy change in these contexts is inherently slow and incremental due to the effect of path dependency [17, 18, 78], the role of the legislative process was not clearly visible in Zimbabwe. This suggests that  informal institutions might carry considerable weight for health reform processes in countries such as Zimbabwe. This might in turn explain why health policy change in some African countries has been attributed to the power and preferences of individuals such as the president and cabinet ministers [79, 80]. Although the power and personal preferences of those in the executive is often viewed negatively as in the case of South African President’s Thabo Mbeki’s resistant stance for public rollout out of Anti-Retroviral Therapy (ART) [81], the experience of Zimbabwe showed that it could be used progressively. For example, in 2002, Zimbabwe’s Minister of Justice, Legal and Parliamentary Affairs declared a period of emergency due to HIV/AIDS pandemic. The move paved way for compulsory licensing for domestic manufacturing of affordable generic ART to facilitate early access to life-saving treatment [82].

From a PEA perspective, one striking feature we found was that unlike PHC, UHC did not feature as ‘high politics’ in Zimbabwe particularly at the implementation stage. As a result, Zimbabwe stands out in contrast with other African countries such as Kenya, Nigeria, Rwanda and Ghana that took bold reform steps to establish pooled, publicly funded  and pre-payment health financing  mechanisms, albeit with several challenges [83–85]. Whilst ‘lack of resources’ is often cited as a reason for slow implementation of such reforms in Zimbabwe, experience from countries such as Ethiopia demonstrated that progress towards UHC could be realized with minimal resources in what has been dubbed ‘Good Health at Low Cost’ [86]. Country experience also reflects the diversity of pathways to achieve UHC relative to own political economy contexts, including initiatives such as Community Based Health Insurance (CBHIs) in Rwanda [87]. By adopting a comparative lens between  PHC and UHC  reforms, we attribute the slow pace of UHC reforms (in particular establishment of publicly funded  and pooled financing mechanisms which anchor UHC principles) to various reasons in line with the determinants of political prioritization put forward by Shiffman and Smith [88]. First, the problems which were a priority during the 1980s such as racial imbalances and rural-urban disparities had clear indicators of severity and carried an emotive societal frame. We posit that those issue characteristics facilitated a high degree of resonance with the political context. Second, clear solutions existed for example ‘ensuring that every citizen resides within a 10 km radius from the nearest clinics’ [89]. In contrast, problems such as lack of  financial protection and catastrophic expenditure under UHC require a technical interpretation. Additionally, the problems under UHC tend to be framed at individual level with limited emotive and political appeal. Relatedly, solutions for achieving UHC such as establishment of pre-paid financing mechanisms are vulnerable to indefinitely remain on the policy agenda without progressing to the implementation agenda because of the perceived technical challenges such as ‘lack of resources’. Third, we found that some policy priorities emerged in the last four decades to the extent that the health sector in general needed to actively compete for attention with other priorities. In particular, from 2000, policies on land reform, agricultural productivity and indigenization have gained a prominent seat in ‘high politics’ including dominating successive electoral manifestos [90]. However, this did not imply that decision makers neglected health care. Rather, it implied that decision makers were influenced by the domestic political economy characteristics of the day when allocating resources between health care and other emerging priority areas. Table 2 below provides a comparison  of political economy variables between Zimbabwe and other settings.

**Table 2:** A comparison of political economy variables between Zimbabwe and other settings

Explanatory variable	Zimbabwe	Other settings/developed countries
**Ideas/Ideology**	-Non-portrayal of polarized ideologies -Influence of external factors on ideology -Ideology sponsors are generally non-visible	-Left-right wing ideologies -Home grown ideologies /crafters of ideologies that diffuse to developing countries -Ideology sponsors can be visible
**Interests**	-Centralized system (power is concentrated in the state) -Health financing reforms not ‘high politics’ for electoral cycles -Non-visible contestation of power -No clear winners and losers -Non-organized interests -Limited role of strategic coalitions and constituency mobilization -Role of external factors/agencies (donors, global health discourses, WHO)	-Pluralistic system (power is dispersed amongst actors) -Health financing reforms a subject of ‘high politics’ for electoral cycles -Visible power contestation -Clear winners and losers -Organized interests -Strategic coalitions and constituency mobilization -Limited role of external factors
**Institutions**	-Influence of informal institutions -Non-legislative reform process -Limited veto points -Reform process framed within the adoption of globally instituted policies/concepts and commitment to regional and international treaties and statutes (e.g., SDGs, UHC, Abuja declaration) -Swift policy reform possible	-Policy reform predominantly through formal institutions -Reform through legislative process -Multiple veto points -Reform process framed within domestic interests -Generally slow reform process

### Implications for policy and research

One important finding from this study is that the current ideas being emphasized as ‘urgent’ in the context of UHC like the establishment of the National Health Insurance have been in the policy domain since the 1980s. This underscores that the political economy factors that slow down UHC reforms are not rooted in the ambiguities of ideas, instead the fundamental issue is how to move from rhetoric to action by aligning ideas, interests and institutions. This is  an inherently political and redistributive process that requires political commitment. At a scholarly level, we posit that the portrayal of UHC as a non-political issue has biased research interest towards the technical aspects of the concept (ideas). What is conspicuously lacking is the analysis of how interests and institutions could favor or hinder the implementation of such ideas. Empirical studies are therefore needed to understand how the interaction of these factors influence the policy process to prospectively gauge the feasibility of desired UHC reforms. Regarding the differences between the political economy variables of Zimbabwe and other settings, policy makers and researchers working in Zimbabwe should go beyond the formal or open policy characteristics that are often portrayed in other settings and explicitly consider the role of informal institutions.

## Conclusion

Health financing reforms in Zimbabwe have been historically influenced and continue to be influenced by political economy dynamics. These factors are often ignored within the domestic UHC discourse. Momentous policy windows such as the political transition to majority rule in 1980 allowed problems and policies related to health care to be framed and treated as political issues which led to marked progress towards universal health care during the first decade of independence under the PHC concept. Over the years, such progress has been severely challenged by shifting political economy realities characterized by the imposition of user fees, episodes of cumulative socio-economic decline and limited resonance of contemporary UHC principles with the political contexts. Despite the challenges, the domestic pioneering of innovative financing models such as the National AIDS Trust Fund is a demonstration that under certain conditions, political windows could momentarily open to advance pro-UHC policies in Zimbabwe. To steer the necessary UHC reforms, actors involved in such efforts should not consider politics as an unhelpful intrusion into their technical work, instead a political economy lens should be explicitly considered to improve the feasibility of reform implementation.

## Data Availability

The datasets during and/or analyzed during the current study available from the corresponding author on reasonable request.
